# Complete genome sequence and methylome analysis of *Dermacoccus nishinomiyaensis* strain 1-2 isolated from sushi

**DOI:** 10.1128/mra.00916-26

**Published:** 2026-10-07

**Authors:** Axel Soto-Serrano, Lukasz Krych, Jørgen J. Leisner

**Affiliations:** 1 Department of Food Science, Faculty of Science, University of Copenhagen 87017, Frederiksberg, Denmark; 2 Department of Veterinary and Animal Sciences, Faculty of Health and Medical Sciences, University of Copenhagen 4321, Frederiksberg, Denmark; Nanchang University, Nanchang, Jiangxi, China

**Keywords:** *Dermacoccus*, sushi, DNA methylation, antiphage systems

## Abstract

*Dermacoccus nishinomiyaensis* is an actinomycete whose isolation from diverse environments and potential for opportunistic pathogenicity have been documented. Here, we report the complete genome sequence of *D. nishinomiyaensis* 1-2, isolated from sushi. We provide annotation statistics and insights, such as the antiphage systems arsenal of the strain, including its methylome.

## ANNOUNCEMENT

*Dermacoccus nishinomiyaensis* (1) is a human-skin commensal (2), but it has also been isolated from an indoor track facility (3), wood ash (4), water for saké brewing (2), salt for Spanish dry-cured ham (5), and the gut of adult flesh flies (6). Although uncommon, opportunistic infections such as catheter-associated bacteremia have been documented (7, 8). This, together with its occurrence in diverse environments, supports the surveillance of *D. nishinomiyaensis* as a contaminant in food and built-environment studies.

Strain 1-2 was isolated from salmon hosomaki sushi, purchased from a restaurant in Copenhagen, Denmark (February 2026), using Plate Count Agar (Oxoid, UK) for enumeration and subcultured on Tryptone Soya Agar (Oxoid, UK), incubating aerobically at 30°C. Genomic DNA was extracted from a single-colony overnight 30°C Tryptone Soy Broth culture after centrifugation (4,402× *g*, 10 min; 12,000× *g*, 3 min) using the Bead-Beat Micro AX Gravity kit (A&A Biotechnology, Gdynia, Poland) and quantified using Qubit 4 with the 1× dsDNA HS kit (ThermoFisher, USA). The DNA was not sheared, and libraries were prepared using the SQK-NBD114.96 Native Barcoding Kit (Oxford Nanopore Technologies, UK), enriching DNA fragments >3 kb with the Long Fragment Buffer. Sequencing was performed on a PromethION 2 Solo using an R10.4.1 Flow Cell (FLO-PRO114M, Oxford Nanopore Technologies, UK). Basecalling employing the dna_r10.4.1_e8.2_400bps_sup@v5.2.0 model with the v5.2.0_4mC_5mC@v1 and v5.2.0_6mA@v1 modified base models, demultiplexing, and adapter trimming were performed with Dorado v1.0.2 (https://github.com/nanoporetech/dorado).

For bioinformatic analyses, all software used default parameters, unless otherwise specified. Hybracter v0.9.0 (9) using the flye --nano-hq model (10) and the Plassembler (11) plsdb_2023_11_03_v2 database was employed for genome assembly and circularization, filtering reads at a minimum quality of 16, a length of 1,000 bp, and subsample depth 110×. After filtering, 29,901 reads with N50 20,498 bp were utilized for the assembly. The genome was reoriented at *dnaA* using dnaapler v0.8.1 (12). Assembly quality was assessed using CheckM v1.2.4 (13) (Table 1). GTDB-Tk v2.3.2 (database release 214) (14) assigned the genome to *D. nishinomiyaensis* (98.91% average nucleotide identity to the type strain). Genome-wide annotation was conducted using Bakta v1.9.4 (15).

**TABLE 1:** Genome assembly and annotation statistics

Genome feature	Value
Genome size (bp)	3,223,617
Chromosome size (bp)	3,049,825
Contigs	3
N50 (bp)	3,049,825
G + C content (%)	68.7
Genome coverage (×)	89
Completeness (%)	100.0
Contamination (%)	0.54
Strain heterogeneity (%)	0.0
Coding density (%)	89.0
Coding sequences	2,924
Transfer RNAs	52
Transfer-messenger RNAs	1
Ribosomal RNAs	9
Non-coding RNAs	2
Non-coding RNA regions	16
Pseudogenes	25
Hypotheticals	370

**Fig 1:**
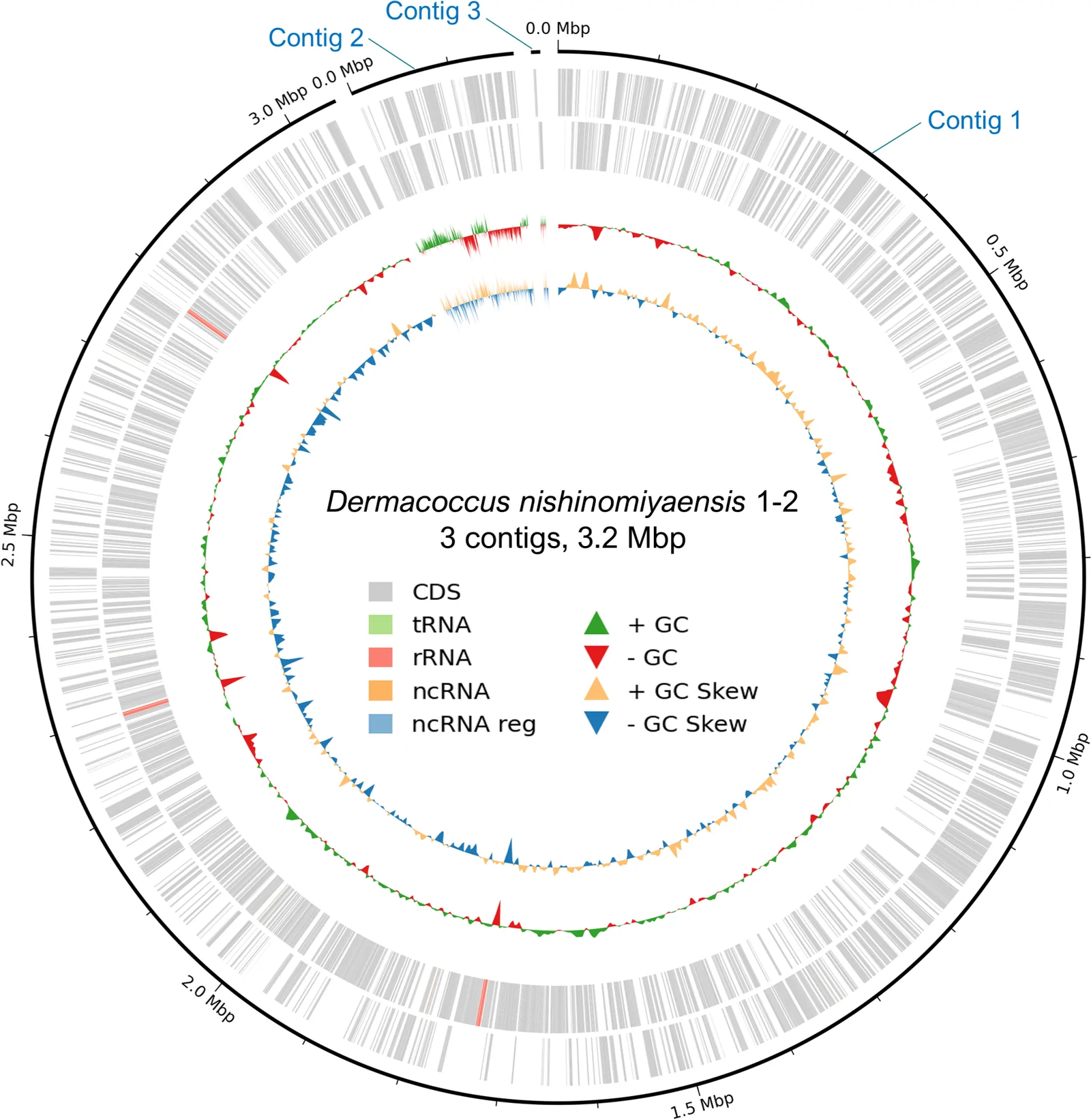
Circular representation of the *Dermacoccus nishinomiyaensis* 1-2 genome. The three contigs are arranged consecutively and separated by gaps. From the outside inward, the rings show genome coordinates, coding sequences on the forward and reverse strands, annotated RNA genes, GC content relative to the genome average, and GC skew. Positive and negative deviations in GC content and GC skew are indicated by the colors shown in the legend.

The genome contains three circular contigs (Fig. 1), including the chromosome, a megaplasmid (165.5 kb), and a circularized mobile element (8.2 kb). PADLOC v2.0.0 (16) predicted several megaplasmid-encoded antiphage systems, including a type V CRISPR-Cas, a type II restriction–modification (RM), Gabija, and an AbiE system. The chromosome encoded a CRISPR array, two PD-T4-6, two SoFic, a dXTPase-based system, and systems based on DNA methylation, including a type III RM, a BREX, and unspecified “DMS” systems. Nanomotif v1.1.2 (17) analysis following its documentation (https://nanomotif.readthedocs.io/en/latest/) identified four putative N6-methyladenine (6mA) methylated motifs: AAG6mAG, CCGA6mAG, CGAA6mAG, and CTGC6mAG.

The 8.2 kb contig encoded small multidrug-resistance transporters potentially associated with tolerance to quaternary ammonium compounds and mobility-associated genes, including a transposase and a relaxase.

Antimicrobial resistance and virulence-associated genes were screened using PathoFact2 v2.0 (18) and ABRicate v1.4.0 (https://github.com/tseemann/abricate) against VFDB (19) using 70% sequence-identity and coverage thresholds. After manual evaluation, no convincing antimicrobial-resistance genes or classical virulence determinants were identified.

## Data Availability

The genomic and DNA read data announced here are available at the National Center for Biotechnology and Information with RefSeq accession number GCF_059774085.1 under the BioProject PRJNA1496822 and BioSample SAMN61732068. The sequencing reads used for the assembly are deposited in the Sequence Read Archive with accession number SRR39683018.
